# Supplementary material for: Angiotensin II Influences Pre-mRNA Splicing Regulation by Enhancing RBM20 Transcription Through Activation of the MAPK/ELK1 Signaling Pathway
Source: Int J Mol Sci. 2019 Oct 12;20(20):5059. doi: 10.3390/ijms20205059 (PMC6829565; doi:10.3390/ijms20205059)
Supplement: Supplementary file 1 [file ijms-20-05059-s001.pdf]

Supplementary

Table S1. Primers used in RT-PCR and QPCR.

**Table S1**	**Primers used in RT-PCR and Qpcr**	
Region/Gene	Primer sequence (5'-3')	Usage
Exon 7-10	F: ACGGTGTCCAGGAGCAAGT	RT-PCR
	R: TTGGCTTTGGTTGCGGAGA	
Exon 10-14	F: TGCCAAAGGTAGTGATCTCCG	RT-PCR
	R: GTGGTCTGCTGAGCATAGGAT	
Exon 54-56	F: CTGTTACCTTACAAGCTGCCGTG	RT-PCR
	R: CCTTAAACCAGGAAACCCTCATG	
Exon 66-68	F: GAAAATGTCACCACCGTCTTG	RT-PCR
	R: TCTCCTGCTACATTGGACACC	
Exon 362-364	F: TGTTCAGCTACAGCTTCCTT	RT-PCR
	R: AGTCAGATCCAAATTCATTCCC	
Ldb3	F: TCCAAGCGTCCTATCCCCATC	RT-PCR
	R: TGTATTCTGTCCCGGTCATCTG	
Camk2g	F: CAACGGTCAACAGTGGCATCC	RT-PCR
	R: GTGTAGGCCTCAAAGTCCCCA	
Trdn	F: CAGAGACAAAGATGGCAGCAA	RT-PCR
	R: CTTCCAGTGATGGTGTTGACA	
Exon 49-50	F: CTCAAACGGCAATGGGAAAG	qPCR
	R: CAGGTGAATTTGGCTAGGTG	
Exon 50-219	F: CAACGAGTATGGCAGTGTC	qPCR
	R: CTTTTCAGCACCACCTCCT	
Exon 108	F: GCATACAGAACATCGTGGTG	qPCR
	R: CTTTGTACCAGGTGACGATG	

Table S2. Primers used to construct report gene.

**Table S2**	**Primers used to construct report gene**
report gene	Primer sequence (5'-3')
pGL3-Rbm20-WT	F: GGGGTACCGGGGAAATTGACACAAAGTCAGGA
	R: GTCCAAGCTTATGGCTACTGCCAGCACCATGC
BS1-Del	F: GGGGTACCGGGGAAATTGACACAAGGAGGGAC
	R: GTCCAAGCTTATGGCTACTGCCAGCACCATGC
BS2-Del	F1: GGGGTACCGGGGAAATTGACACAAAGTCAGGA
	R1: CGTCTCTGGAAATTTAAAGAGAGTTCACCGACTC
	F2: GAGTCGGTGAACTCTCTTTAAATTTCCAGAGACG
	R2: CATCGCCAGCGCGAAGTGCGCCCGGGGCTTGGCATCCTCCTGCGGCTGGAGCCCAGC CAGCGGACCCGACAC
	F3: CCAAGCCCCGGGCGCACTTCGCGCTGGCGATGTCCTCGGGACTCTGCCGGAGCCGGG CAGCCTGTGAGCACTACAAGG
	R3: CAGGGGGCGTCCCCGGGGCGCGAGACGCCCAG
	F5: CTGGGCGTCTCGCGCCCCGGGGACGCCCCCTG
	R5: GTCCAAGCTTATGGCTACTGCCAGCACCATGC
